# A Fhit-mimetic peptide suppresses annexin A4-mediated chemoresistance to paclitaxel in lung cancer cells

**DOI:** 10.18632/oncotarget.9179

**Published:** 2016-05-04

**Authors:** Eugenio Gaudio, Francesco Paduano, Apollinaire Ngankeu, Francesco Ortuso, Francesca Lovat, Sandra Pinton, Sabrina D'Agostino, Nicola Zanesi, Rami I. Aqeilan, Pietro Campiglia, Ettore Novellino, Stefano Alcaro, Carlo M. Croce, Francesco Trapasso

**Affiliations:** ^1^ Department of Molecular Immunology, Virology and Medical Genetics, The Ohio State University, Columbus, Ohio, USA; ^2^ Lymphoma & Genomics Research Program, IOR Institute of Oncology Research, Bellinzona, Switzerland; ^3^ Dipartimento di Medicina Sperimentale e Clinica, University Magna Græcia, Campus “S. Venuta”, Catanzaro, Italy; ^4^ Dipartimento di Scienze della Salute, University Magna Græcia, Campus “S. Venuta”, Catanzaro, Italy; ^5^ The Lautenberg Center for Immunology and Cancer Research, Institute for Medical Research, The Hebrew University, Jerusalem, Israel; ^6^ Dipartimento di Farmacia, Università di Salerno, Fisciano, Italy; ^7^ Dipartimento di Farmacia, Università degli Studi di Napoli “Federico II”, Napoli, Italy

**Keywords:** fragile histidine triad, FHIT, annexin A4, ANXA4, mimetic peptides

## Abstract

We recently reported that Fhit is in a molecular complex with annexin A4 (ANXA4); following to their binding, Fhit delocalizes ANXA4 from plasma membrane to cytosol in paclitaxel-resistant lung cancer cells, thus restoring their chemosensitivity to the drug. Here, we demonstrate that Fhit physically interacts with A4 through its N-terminus; molecular dynamics simulations were performed on a 3D Fhit model to rationalize its mechanism of action. This approach allowed for the identification of the QHLIKPS heptapeptide (position 7 to 13 of the wild-type Fhit protein) as the smallest Fhit sequence still able to preserve its ability to bind ANXA4. Interestingly, Fhit peptide also recapitulates the property of the native protein in inhibiting Annexin A4 translocation from cytosol to plasma membrane in A549 and Calu-2 lung cancer cells treated with paclitaxel. Finally, the combination of Tat-Fhit peptide and paclitaxel synergistically increases the apoptotic rate of cultured lung cancer cells and blocks *in vivo* tumor formation.

Our findings address to the identification of chemically simplified Fhit derivatives that mimic Fhit tumor suppressor functions; intriguingly, this approach might lead to the generation of novel anticancer drugs to be used in combination with conventional therapies in Fhit-negative tumors to prevent or delay chemoresistance.

## INTRODUCTION

The *FHIT* gene encompasses the most common fragile site, *FRA3B* at 3p14.2 [1, 2] in human lymphoblasts; interestingly, although this region is not the most fragile locus in epithelial cells [3], *FHIT* loss is commonly reported in human tumors of this derivation as an early event [4], not only through genetic inactivation (i.e., deletions and translocations) [5] but also through the silencing of its promoter due to hypermethylation [6].

Several evidences pointed at *FHIT* as a tumor suppressor gene; in fact, its genetic ablation in mice results both in an increase of spontaneous tumors and in a much higher susceptibility to develop carcinogen-induced tumors than wild-type counterparts [7]. Moreover, *FHIT* overexpression driven *in vivo* through recombinant viral vectors, prevented tumor development in *Fhit* knock-out mice [8, 9]. Furthermore, *FHIT* restoration in *FHIT*-negative cancer cells of both epithelial origin and leukemias blocks *in vivo* tumor formation and triggers caspase-mediated apoptosis [10–12].

*FHIT* is one of the genes mostly involved in human tumorigenesis; however, regardless of the efforts, Fhit function still remains largely obscure. *FHIT* encodes for an enzyme whose *in vivo* substrate has not been identified yet; however, in cell free assays its product hydrolizes diadenosine polyphosphate substrates, preferentially diadenosine triphosphate (Ap3A) but also diadenosine tetraphosphate (Ap4A) [13, 14]. The design of mutant alleles of *FHIT* proved that only mutants still binding the substrate could, at least partially, retain the ability to trigger apoptosis of Fhit-negative cancer cells [15, 16]. Only recently, we begun to shed lights about the molecular pathways involving Fhit protein activity. In fact, Hsp60 and Hsp10 take Fhit protein into the mitochondrion where it binds some mitochondrial proteins, including Fdxr; this protein complex induces the generation of reactive oxygen species (ROS) which, in turn, triggers apoptosis of Fhit-negative cancer cells [17]. As Fhit protein is virtually ubiquitously distributed into the cell [17, 18], later on we tried to isolate novel Fhit partners with oncogenic activity starting from cell membranes; we successfully identified annexin A4 (ANXA4) [19], a protein belonging to a superfamily of calcium-regulated and phospholipid membrane-binding proteins [20]. ANXA4 is overexpressed in various tumors [21–23] and it is involved in chemoresistance [21, 22, 24–27] other than invasion and metastasis [28]. Intriguingly, Fhit restoration in Fhit-negative lung cancer cells blocks ANXA4 translocation from cytosol to the inner side of plasma membrane during paclitaxel administration, an effect that contribute to chemoresistance, thus sensitizing again cancer cells to the drug [19].

In this study, we report that a short Fhit-derived peptide still able to physically bind ANXA4, recapitulates the Fhit wild-type activity with regard to ANXA4, as this Fhit sequence can prevent ANXA4 translocation to plasma membrane in paclitaxel-treated lung cancer cells, thus resulting in the restoration of chemosensibility to the drug both *in vitro* and *in vivo* experiments.

## RESULTS

### Annexin 4 binds the N-terminus of Fhit protein

**Figure 1:**
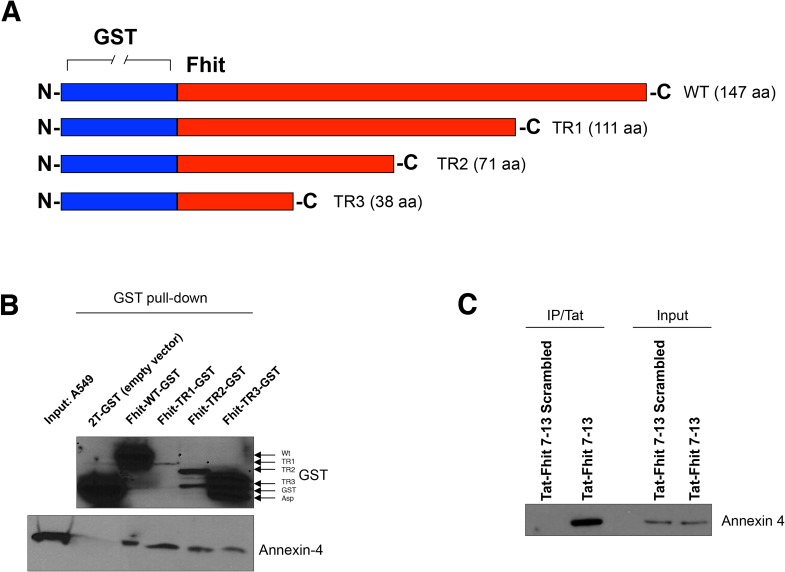
Fhit peptide interacts with Annexin 4 **A.** GST-Fhit fusion protein and three deletion mutant proteins. Fhit was deleted from the C-terminal site. **B.** GST-*FHIT* plasmids were amplified in BL21 bacteria by stimulation with IPTG 0.5 μM for 6 h at 30°C. Recombinant GST-Fhit fusion proteins were purified with GSH resin beads and added to A549 total lysates. 12 h after incubation at 4°C, GSH resin beads were washed and proteins eluted. Proteins were separated on a polyacrylamide gel, transferred to nitrocellulose filters and probed with antibodies raised against Annexin 4 or GST. **C.** A549 cells were treated with Tat-scrambled peptide or Tat-Fhit 7-13 peptide (150 μM) 24 h after Tat-Fhit 7-13 peptide administration, cell lysates enriched in membrane fraction were co-immunoprecipitated with a Tat monoclonal antibody, proteins were separated on a polyacrylamide gel, transferred to nitrocellulose filters and probed with an Annexin 4 antibody. Inputs were run as control for equal immunoprecipitated protein amounts.

In order to map the region of Fhit protein binding Annexin A4, we cloned downstream GST three truncated *FHIT* cDNAs, namely TR1 (332 bp), TR2 (212 bp) and TR3 (113 bp), all containing the N-terminus common to the wild-type Fhit protein. TR1, TR2, and TR3 correspond to the 111, 71, and 38 N-terminal aminoacids of Fhit protein, respectively (Figure 1A). Annexin A4 binds wild-type Fhit/GST fusion protein as well as all shorter forms of Fhit fusion proteins, indicating its binding to the N-terminus of Fhit (Figure 1B). In our previous paper, we reported that Fhit/Annexin A4 interaction was only detectable in the presence of DSP [19]. DSP is a cross-linker containing an amine-reactive N-hydroxysuccinimide (NHS); NHS esters react with primary amine group located at lysine (Lys, K) residue sidechain. Since Lys residues are abundant and easily accessible on the hydrophilic surface of most proteins they usually crosslink with high efficiency.

We assumed that the Lys interacting to DSP must be positioned on a Fhit surface region binding Annexin A4. Due to the unavailability of experimental structures reporting such a complex, molecular modeling studies were performed to highlight the most favored Lys residues for interacting with DSP (Supplementary Figure S1). Taking into account the reaction between the Lys and the cross-linker, we have considered two different molecular descriptors in order to predict the most favored adduct formation: a) the exposition of the nucleophilic residue, and b) the electron availability on the primary amine located at the Lys sidechain. The first descriptor was computed as the average solvent surface area (ASASA) related to the Lys sidechain and the second was derived from the residue partial charge distribution. Both parameters required to take into account the Fhit conformation flexibility; consequently extensive molecular dynamics (MD) simulations were performed on our Fhit 3D model sampling, at regular time intervals, 150 structures. These conformers were used for the ASASA calculation and, after clusterisation, for the quantum mechanics computing of the Mulliken charge distribution (Supplementary Figures S2-S3). Moreover, with the aim to deep evaluate Fhit conformational properties, in particular highlighting the most flexible protein areas, MD trajectory was submitted to residue fluctuation analysis (RFA) (Supplementary Figure S4). The results marked the Fhit 108-127 loop as having greater flexibility than the remainder of the protein. Taking into account its experimental activity, we focused our attention on structural similarities between the Fhit peptide segment 1-38 and the full length protein, by submitting the short peptide to the same MD protocol adopted for Fhit. The MD trajectories of 1-38 and Fhit were compared by computing, frame by frame, the root mean square deviation (RMSd), generating a squared matrix containing 22500 RMSd values (corresponding to all possible combination among the 1-38 and Fhit MD structures). Such an analysis revealed remarkable similarities, with ~81% of the structures reporting an all atoms RMSd value ranging from 2 to 4 Å (Supplementary Figure S5). To identify the region of 1-38, containing Lys residues (11, 18 and 29), structurally closest to Fhit, the short peptide MD trajectory was submitted to the same RFA previously described for the full length protein. The comparison between 1-38 and Fhit RFA revealed the stronger analogies for the region containing Lys11 (Supplementary Figure S6). In particular His8, Leu9, Ile10, Lys11 and Pro12 of 1-38 peptide reported an average difference in terms of RMSd of about 0.02 Å with respect to the full-length Fhit.

On the basis of the above reported information, we addressed to the Fhit 8-12 residue sequence a key role in the Annexin 4 recognition. With the aim to improve water solubility of the new shorter peptide, we included hydrophilic residues at Fhit position 7 (Gln) and 13 (Ser).

To validate this hypothesis, we tested a synthetic Fhit 7-13 peptide on A549 and Calu-2 cultured lung cancer cells. To assure the cell permeation of Fhit 7-13 peptide, the sequence was fused downstream to a Tat peptide (an epitope mapping at position 47-58 of the HIV Tat protein); 24 h after Tat-Fhit 7-13 peptide administration, cell lysates were co-immunoprecipitated with a Tat monoclonal antibody. As demonstrated in Figure 1C, Annexin 4 was detectable only in the presence of Fhit 7-13 peptide, but not in cells treated with a Tat-Fhit 7-13 scrambled peptide.

### Fhit peptide blocks annexin 4 translocation from cytosol to plasma membrane

**Figure 2:**
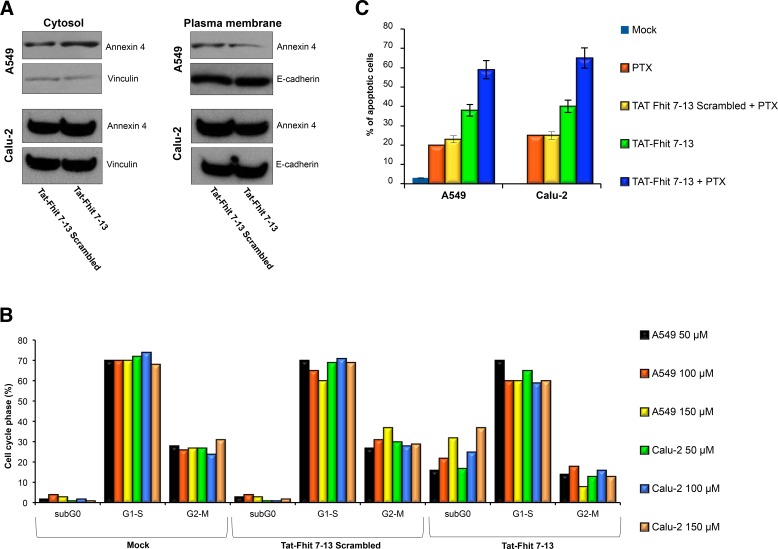
Tat-Fhit 7-13 peptide blocks Annexin 4 translocation from cytosol to plasma membranes, triggers apoptosis, and sensitizes lung cancer cells to paclitaxel **A.** A549 and Calu-2 lung cancer cells were treated with Tat-scrambled peptide, Tat-Fhit 7-13 peptide (150 μM) and 800 nM paclitaxel for 24 h. Proteins from cytosolic and membrane fractions were separated on a polyacrylamide gel, transferred to nitrocellulose filter, and probed with Annexin 4 antibody. Vinculin and E-cadherin were used to normalize protein loading of cytosolic and plasma membrane proteins, respectively. **B.** A549 and Calu-2 cells were untreated or treated with 50, 100 and 150 μM Tat-scrambled peptide or Tat-Fhit 7-13 peptide and then 24 h later evaluated by flow cytometric analysis; a representative experiment is reported. **C.** A549 and Calu-2 cells treated with Tat-scrambled peptide and Tat-Fhit 7-13 peptide for 24 h, as described in B, were analyzed by TUNEL assay; mean values ± SD of three experiments are reported.

As previously reported [19], treatment of A549 and Calu-2 cancer cells with paclitaxel induced cytosolic depletion of Annexin A4 that underwent a considerable translocation to the inner side of plasma membrane. Fhit overexpression blocked Annexin A4 translocation from cytosol to the plasma membrane, observed after paclitaxel administration. Here, we tested the ability of the Tat-Fhit 7-13 peptide to block Annexin 4 in the cytosol. Figure 2A shows that the simultaneous treatment with paclitaxel and Tat-Fhit 7-13 peptide, was able to block Annexin A4 translocation from cytosol to plasma membrane compared to the scrambled Tat-Fhit 7-13 used as a control in both A549 and Calu-2 cells. In addition, other peptides designed from both N- and C-termini of the Fhit protein, failed either to bind or sequester Annexin 4 in the cytosol (data not shown). These data indicated that the N-terminus of Fhit is responsible for the binding with Annexin 4 and, more specifically, that the peptide sequence ranging from position 7 to 13 of Fhit is crucial for the binding.

### Tat-Fhit 7-13 peptide triggers apoptosis of lung cancer cells and sensitizes them to paclitxel

To determine if Tat-Fhit 7-13 peptide could mimic the effects of the wild-type Fhit protein on A549 and Calu-2 lung cancer cell lines, cells were treated with a single administration of 50, 100 or 150 μM Fhit Tat-7-13 peptide and 24 h later cell death was assessed by flow cytometry. Interestingly, we observed dose-dependent sub-G1 peaks accounting for 16%, 22%, and 32% of cell population, for the growing doses of peptide administered to A549 cells. The Tat-Fhit 7-13 scrambled peptide had no effect in triggering cell death (Figure 2B). Furthermore, Tat-Fhit 7-13 peptide sensitizes A549 cells to paclitaxel, as shown by TUNEL assay; in fact, the percentage of apoptotic cells increased three-fold compared to controls (60% *versus* 20%) (Figure 2C). Comparable results were obtained after treatment of Calu-2 cells with Tat-Fhit 7-13 peptide alone or in combination with paclitaxel (Figure 2B and 2C).

Taken together, these data indicate that the Fhit 7-13 peptide can partly recapitulate wild-type Fhit activity in triggering apoptosis of A549 and Calu-2 lung cancer cells.

### Tat-Fhit 7-13 peptide in combination with paclitaxel blocks *in vivo* tumor formation

We tested the stability of our peptides in the culture medium over adherent cells through Mass Spectroemtry-MALDI. Twenty-four hours after their administration to the medium, both scrambled and 7-13 Fhit peptides resulted still stable and undegraded (see Supplementary Figure S7A-S7C).

**Figure 3:**
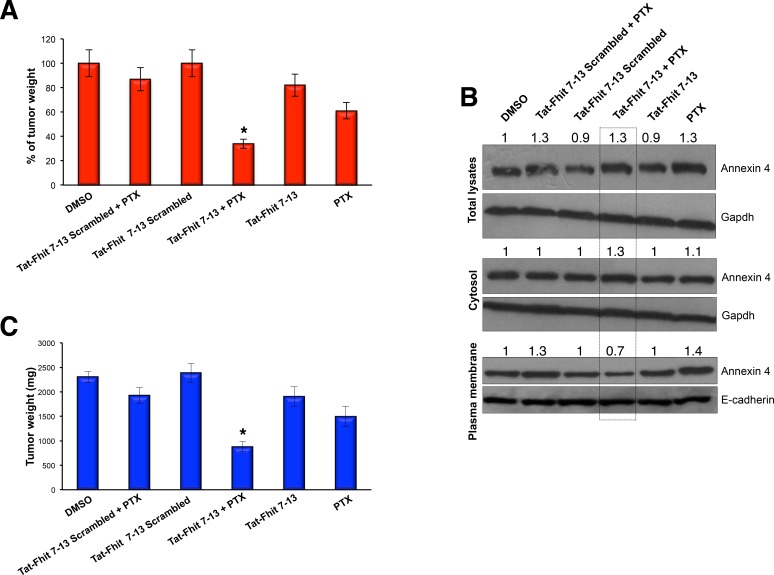
Tat-Fhit 7-13 peptide in combination with paclitaxel blocks *in vivo* tumor formation **A., C.** Nude mice were subcutaneously injected with 1.5 × 10^7^ A549 cells. When tumors reached 15 mm diameter, mice were mock-treated, treated paclitaxel (single IV administration of 40 mg/kg), with scrambled Tat peptide (50 mg/kg) alone or in combination with paclitaxel, with Tat-Fhit 7-13 peptide (50 mg/Kg) alone or in combination with paclitaxel; paclitaxel and peptides were injected in a single administration and mice were monitored on a regular basis. Two days later, mice were sacrificed and tumors were evaluated by percent **A.** and by weight **C.**. Bar graphs show mean ± SEM for values from 5 mice (* *P* < 0,05). **B.** Tumor xenografts excised from mice were lysed, proteins from cytosolic and membrane fractions were separated on a polyacrylamide gel, transferred to nitrocellulose filter, and probed with Annexin 4 antibody. Gapdh and E-cadherin were used to normalize protein loading of cytosolic and plasma membrane proteins, respectively. The numbers above the blots indicate the intensity of the band expressed as ratio “gene product ANNEXIN 4/GAPDH or E-CADHERIN” and normalized to “DMSO”.

We further investigated the *in vivo* effects of Tat-Fhit 7-13 peptide with or without paclitaxel treatment in a preclinical model of lung cancer, using six groups of mice (*n* = 5 mice per group). Each group was subcutaneously injected with 1×10^7^ A549 cells. When tumors reached the diameter of 15 mm, three groups were treated either with a single administration of 40 mg/kg paclitaxel, or 50 mg/kg Tat-Fhit 7-13 peptide, or both; mice were monitored on a regular basis. The remaining three groups, used as controls, were either left untreated or treated with Tat-Fhit 7-13 scrambled or treated with Tat-Fhit 7-13 scrambled plus paclitaxel; 48 h after treatments, mice were sacrificed and tumors were evaluated by weight. A dramatic effect on tumor growth inhibition was observed in the group of mice treated with both paclitaxel and Tat-Fhit 7-13 peptide compared to Tat-Fhit 7-13 or paclitaxel alone or controls. Tumors treated with Tat-Fhit 7-13 or paclitaxel showed a reduction in tumor wt to 82% or 60% of controls, respectively, while their simultaneous administration resulted in tumor reduction to 33% of controls (Figure 3A, 3C, and Supplementary Figure S8). After tumor protein lysate fractionation into cytosolic and plasma membrane fractions, we observed that Tat-Fhit 7-13 treatment reduced the extent of Annexin A4 translocation to the plasma membrane after *in vivo* administration of paclitaxel (Figure 3B), thus indicating that the reported effect on tumor shrinkage in mice treated with the Tat-Fhit 7-13 plus paclitaxel was mainly due to the ability of the peptide to trap Annexin A4 in the cytosol.

## DISCUSSION

Fhit loss has been extensively linked to many processes involved in human and experimental tumorigenesis, including reduced apoptosis, genomic instability, chemoresistance, and, more recently, invasion [29–31], even though the mechanisms governing its activity are still largely obscure. The contribution of Fhit loss to these hallmarks of cancer [19] makes this small protein a very interesting candidate for the development of novel anticancer therapies. However, as no pathway for Fhit function has been discovered for long time after *FHIT* gene cloning, the only therapeutic approach used to restore Fhit function in cancer cells has been limited to virus-mediated gene therapy of preclinical models of cancer [32].

We recently begun to shed lights about Fhit protein partners unveiling novel pathways depending upon it; in fact, by using a proteomic approach we reported for the first time a Fhit complex including Hsp10, Hsp60, and ferredoxin reductase which underlies and supports the role of Fhit in apoptosis through the generation of free radicals in mitochondria [17]. However, even though there are growing evidences of mitochondria as druggable organelles for cancer therapy [33], Fhit is not suitable to this matter, as it is evident the need of the wild-type protein to restore susceptibility to apoptosis triggered by reactive species of oxygen in Fhit-negative cancer cells [15, 16, 34]. Thus, although encouraging as a proof of principle, the possibility of overexpressing the native Fhit protein through viral vectors is very far from large-scale clinical applications for cancer treatment.

These findings prompted us to search for novel Fhit partners with the purpose to identify proteins with oncogenic activity in order to interfere with their function in cancer cells. The isolation of Annexin A4 as a novel Fhit-interacting protein was a very interesting finding for further studies because of its oncogenic role in the pathogenesis of cancer [19, 28] and chemoresistance, assessed for the first time in renal cancer cells resistant to paclitaxel and then also highlighted with cisplatin in cancer cell lines of different types [35, 36]. Intriguingly, as Fhit loss is also involved in cancer cells refractory to paclitaxel and cisplatin [17, 30], the discovery of the Fhit-ANXA4 complex offers opportunities of investigation to search for novel mechanisms responsible for chemoresistance, an event connected to the failure of pharmacological treatment of cancer patients that accounts for their dismal prognosis.

We recently studied the possible role of Fhit in paclitaxel resistance with regard to ANXA4 overexpression; we demonstrated that, while ANXA4 is overexpressed and located to the inner side of plasma membrane in Fhit-negative lung cancer cells treated with paclitaxel, virally-mediated Fhit overexpression restores sensitivity to paclitaxel both *in vitro* and *in vivo* by delocalizing ANXA4 to cytosol [19]. In the present study, as apparently the interference with ANXA4 could possibly not require a full length Fhit protein, we explored the opportunity to identify the shortest Fhit protein sequence still able to recognize the oncogenic ANXA4 thus exerting the biological effects of the Fhit wild-type protein.

The molecular mapping of the Fhit protein domain interacting with Annexin A4 led to narrow the interaction domain to aminoacids 7 to 13 of the Fhit protein. Interestingly, the introduction of this peptide into cells did mimic the behavior of wild-type Fhit protein toward Annexin A4; in fact, the administration of a synthetic Tat-Fhit 7-13 peptide to lung cancer cells: a) blocked Annexin A4 translocation from cytosol to plasma membrane; b) acted synergistically with paclitaxel in triggering apoptosis of lung cancer cells; c) induced dramatic regression of A549 tumor xenografts in combination with paclitaxel.

Our model suggests that both Fhit protein and Fhit-mimetic peptide could interfere with a protein complex recruited by ANXA4 at the plasma membrane level thus determining its relocalization to cytosol. The clarification of the fine-tuning mechanisms governing the Fhit-ANXA4 binding will be a quite interesting field of investigation for better understanding the role of both Fhit and ANXA4 in chemoresistance.

In conclusion, these findings open the interesting perspective of the generation of an innovative drug to be translated in cancer therapy, as the pursue of a possible approach able to overcome chemoresistance would be desirable to fight otherwise incurable cancers. The Fhit 7-13 peptide might serve as a lead compound for the development of synthetic Fhit-mimetic small molecules with the fascinating perspective of their combination with already commercially available drugs; this approach would apparently be helpful for patient refractory to conventional therapies alone, thus making anticancer therapies less toxic and more effective.

## MATERIALS AND METHODS

### Cell culture and transfection experiments

A549 cells were maintained at 37°C in a humidified atmosphere of 5% CO_2_ in the appropriate growth medium with supplements added as recommended.

### Immunoblotting, GST pull-down and fractionation analysis

Total proteins were extracted with Nonidet P40 (NP-40) lysis buffer; cytosolic and plasma membrane proteins were extracted using the FractionPREP-cell fractionation system (Biovision). Total lysates with enriched plasma membrane proteins, used for both mass spectrometry and immunoprecipitations analyses, were obtained using Mem-PER Eukaryotic Membrane Protein Extraction Kit (Pierce).

For immunoblotting, proteins (50 μg) were separated on polyacrylamide gels and transferred to nitrocellulose filter membranes. Membranes were blocked in 5% non-fat dry milk, incubated with primary anti-Annexin 4, GAPDH, and E-Cadherin antibodies (Santa Cruz Biotechnology), detected by the appropriate secondary antibodies, and revealed by enhanced chemiluminescence (ECL; Amersham Inc.).

Full-length *FHIT* cDNA (441 bp) and shorter cDNAs, *FHIT*-TR1 (332 bp), *FHIT*-TR2 (212 bp), and *FHIT*-TR3 (113 bp) containing the common *FHIT* 5′-end were cloned in the pGEX-2T plasmid, and were expressed in *E. coli* (BL21).

To clone wild-type *FHIT* and the truncated forms, the following primers containing a BamHI restriction site were used:

*FHIT* forward: 5′- GGATCCTCGTTCAGATTTGGCCAA-3′

*FHIT* rev TR1: 5′- GGATCCGTCATTCCTGTGAAAGTCTCCAGCCTT - 3′

*FHIT* rev TR2: 5′-GGATCC-CCCATGGAAATGTTTTTCCACCACTGT-3′

*FHIT* rev TR3: 5′-GGATCC-CACAAGGACATGTCCTGGTACCACAGGTT-3′

PGEX-2T plasmid, *E. coli* BL21, and Glutathione Sepharose 4B resin were from Amersham.

### TUNEL assay

A549 cells were assessed for the induction of single strand breaks (indicative of apoptosis) by the terminal deoxynucleotidyl transferase mediated X-dUTP nick end labeling (TUNEL) assay using the *in situ* cell death detection kit (Boehringer/Roche), according to the manufacturer's recommendations.

### Flow cytometry

A549 cells were collected and washed in PBS solution. DNA was stained with propidium iodide (50 μg/ml) and analyzed with a FACScan flow cytometer (Becton-Dickinson) interfaced a Hewlett-Packard computer. Cell cycle data were analyzed with the CELL-FIT program (Becton-Dickinson).

### Fhit peptides

Tat peptide (from the amino acids 47-58 HIV-1 Tat protein -YGRKKRRQRRR) was synthesized upstream to Fhit peptides in order to drive them into the cell. Both Fhit and scrambled peptides were from Invitrogen. A549 cells were treated with the following peptides: Tat-Fhit 7-13 (Tat-G-QHLIKPS); Tat-Scrambled 7-13 (Tat-G-LSKQPHI). Peptides were administered to cell cultures at the concentrations of 50, 100, and 150 microM; 24 h after treatment, cell cycle perturbations were assessed by flow cytometric analysis [37, 38].

### Animal studies

Mice were maintained and animal experiments conducted under institutional guidelines established for the Animal Facility at The Ohio State University; nu/nu mice were purchased from The Jackson Laboratory. Tumors were established by injecting 1 × 10^7^ A549 cells subcutaneously into the right flanks of 6 wk-old female nude (nu/nu) mice. Paclitaxel was administrated intravenously as a single treatment at the concentration of 40 mg/kg. Synthetic peptides were used *in vivo* at the concentration of 50 mg/kg; mice were injected intravenously with a single dose of peptides and sacrificed 48 h later to evaluate tumor weight.

Tumor size was measured on regular basis by using a digital caliper and the equation V (in mm^3^) = (A × B^2^)/2, where A is the largest diameter and B is the perpendicular diameter.

### Molecular modeling

With the aim to identify a target model for our theoretical studies, the 3D crystallographic structures, available into the Protein Data Bank (PDB) with codes 1EMS, 1-2FHI, 1-6FIT [39–43], reporting Fhit, alone or interacting with ligands or proteins, were selected and graphically inspected (Supplementary Figure S1). The model 4FIT, reporting the lowest number of missing residues and a X-ray resolution equal to 2.5 Å, was selected and considered in our simulation. The original PDB structure required a preliminary treatment consisting in the addition of the missing amminoacid at position 1 and of the highly flexible loop corresponding to the sequence 108-127. Co-crystallized water molecules were removed and hydrogen atoms were added. All preliminary procedures were carried out using the Prime module and the Maestro GUI, as implemented in the Schrodinger software suite 2011 [44]. Shortened Fhit derivatives, sequences 1-38 and 7-13, were obtained from the 4FIT model. All molecular dynamics (MD) simulations were performed using the force field OPLSA-2005, up to 15 ns at 300° K using the version 3.0 of the Desmond program [45] sampling 150 structures at regular time intervals. Water solvent effects were taken into account by means of the explicit solvation model TIP3. Lys sidechains solvent accessible surface areas were computed onto the MD sampled structures considering a water mimicking probe with a radius equal to 1.4 Å. MD trajectories were clustered using the average hierarchical linkage method considering a backbone related root mean square deviation (RMSd) cutoff equal to 2.0 Å among the members of the same cluster. The most representative structure, of each cluster, was submitted to quantum mechanics/molecular mechanics (QM/MM) approach for computing the Mulliken charge distribution related to the Lys sidechains. These residues were treated using the density functional theory (DFT) and the LACVP* basis set, the rest of the target was optimized using the OPLS-2005 force field. QM/MM calculations were performed by means of the version 5.7 of the QSite software [46, 47].

### Statistics

All graph values represent means ± SEM from three independent experiments with each measured in triplicate. The differences between two groups were analyzed with unpaired two-tailed Student's *t* test. *P* < 0.05 was considered statistically significant and indicated with asterisks as described in figure legends.

## SUPPLEMENTARY MATERIAL FIGURES


