# Supplementary material for: A Fhit-mimetic peptide suppresses annexin A4-mediated chemoresistance to paclitaxel in lung cancer cells
Source: Oncotarget. 2016 May 4;7(21):29927–36. doi: 10.18632/oncotarget.9179 (PMC5058653; doi:10.18632/oncotarget.9179)
Supplement: Supplementary file 1 [file oncotarget-07-29927-s001.pdf]

A Fhit-mimetic peptide suppresses annexin A4-mediated chemoresistance to paclitaxel in lung cancer cells

Supplementary Material

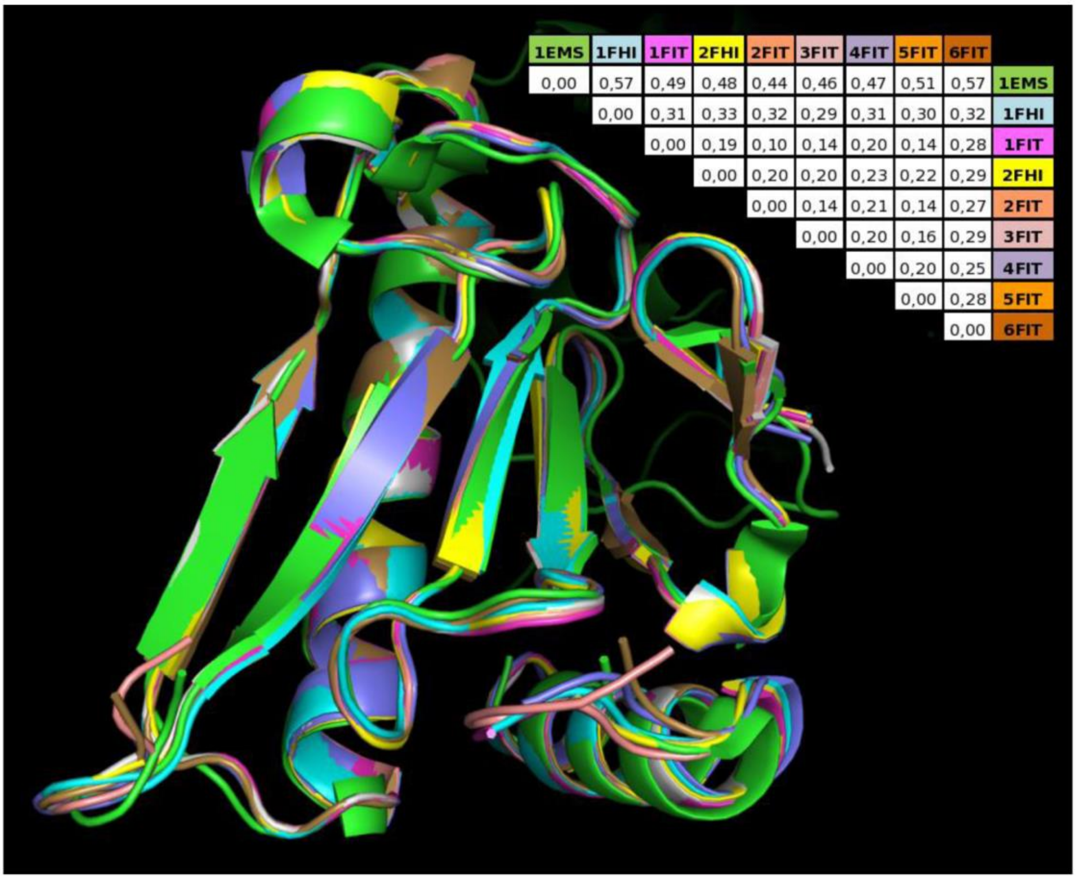

Supplementary Figure S1. Conformation similarity analysis of Protein Data Bank available Fhit structures. Fhit cristallographic models deposited in the Protein Data Bank were aligned and compared one each other by computing the root mean square deviation (in Å).

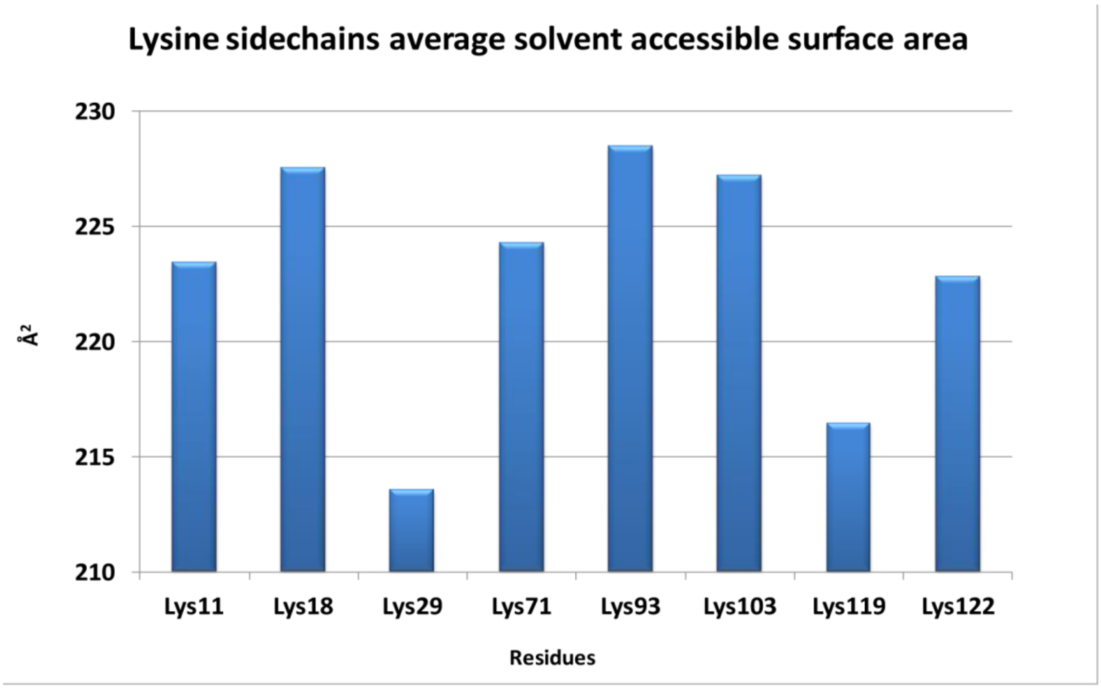

Supplementary Figure S2. Lysine sidechains average solvent accessible surface area. Fhit Lys residues solvent exposition was computed on full length protein molecular dynamics trajectory. The solvent was mimicked by a 1.4 Å radius probe.

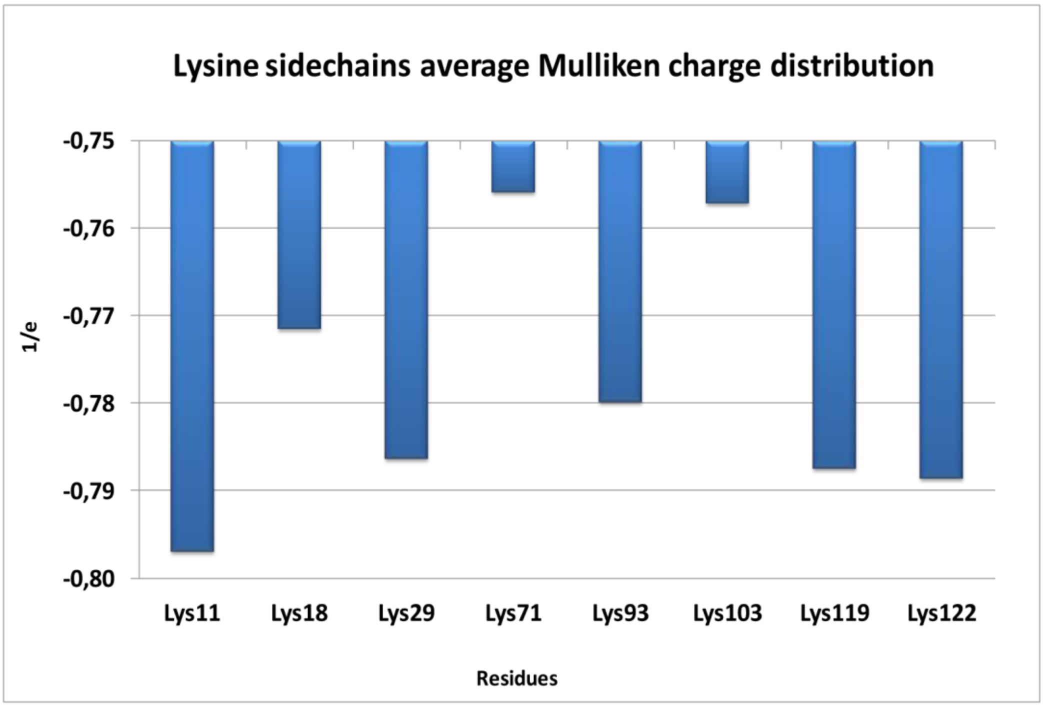

Supplementary Figure S3. Lysine sidechains averageMulliken charge distribution. Charge distribution was adopted as a reactivity descriptor of Fhit Lys residues.

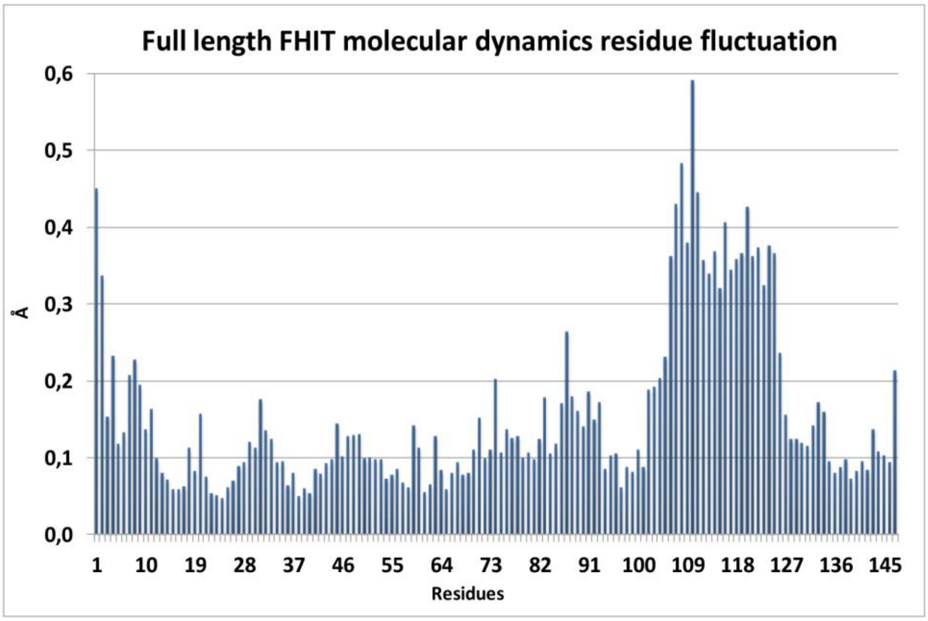

Supplementary Figure S4. Full length Fhit molecular dynamics residue fluctuation. Conformation variability of full length Fhit residues during the molecular dynamics simulation.

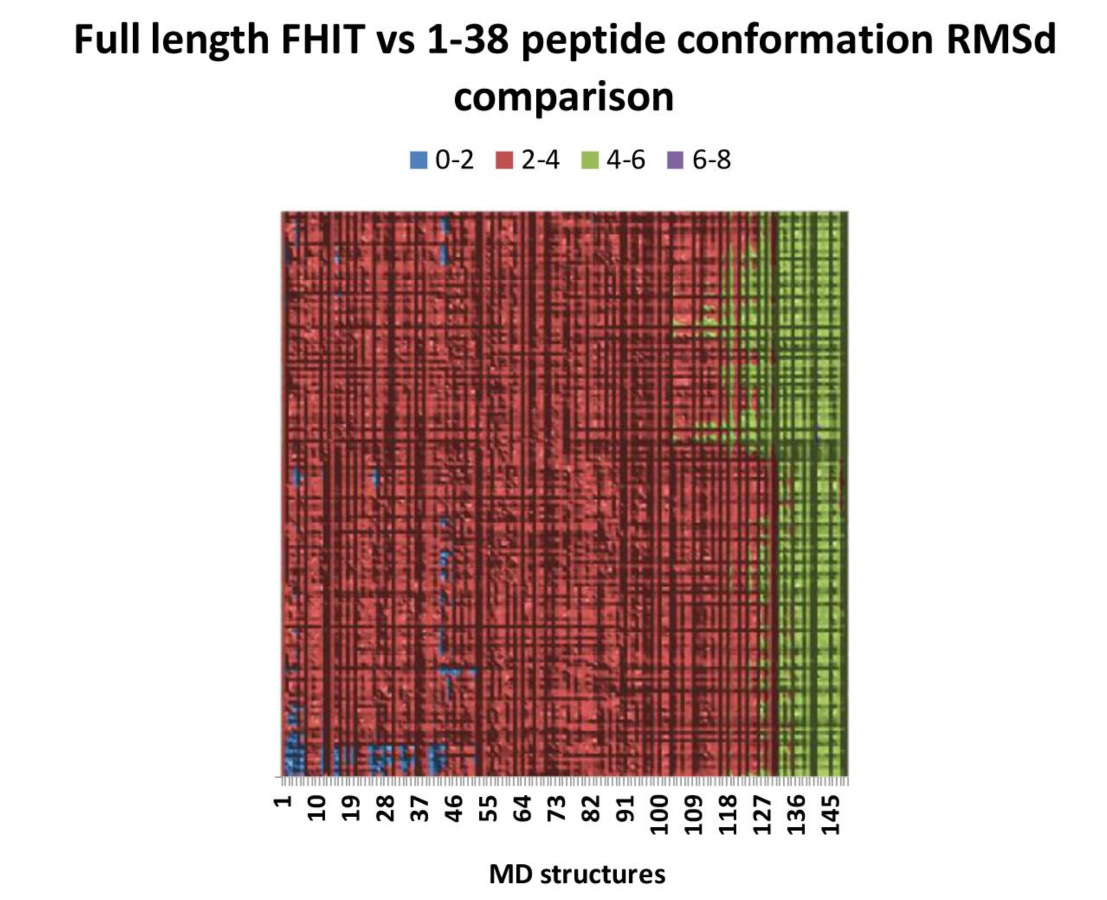

Supplementary Figure S5. Full length Fhit vs 1-38 peptide conformation RMSd comparison. Root mean square deviation values (in Å) computed, frame by frame, on full length Fhit and 1-38 peptide molecular dymanics trajectories.

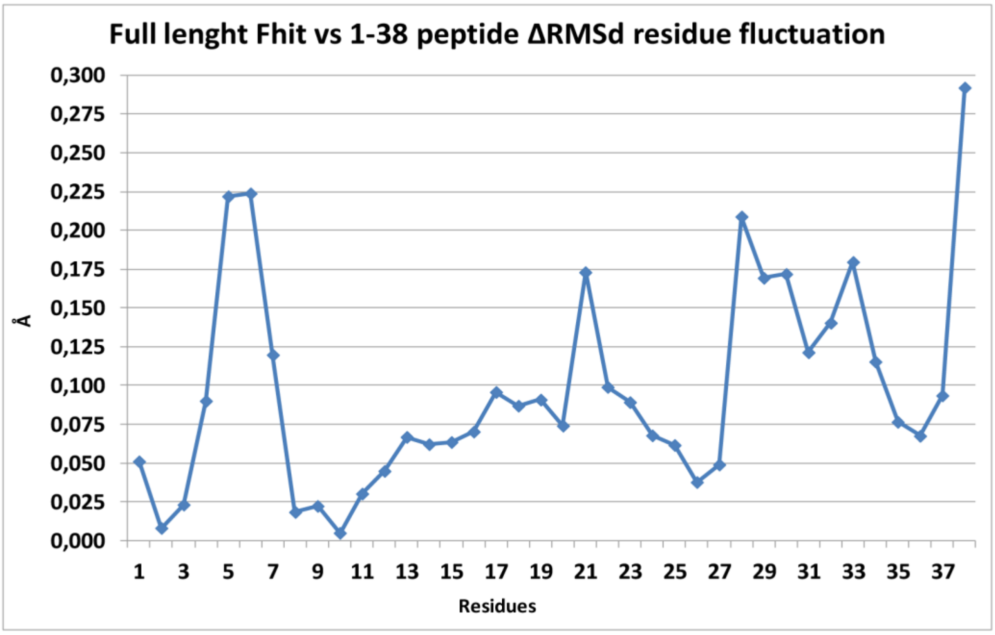

Supplementary Figure S6. Full length Fhit vs 1-38 peptide ΔRMSd residue fluctuation. Difference of 1-38 residues fluctuation in full length and shortened peptide molecular dynamics trajectories.

A

7-13 Fhit peptide

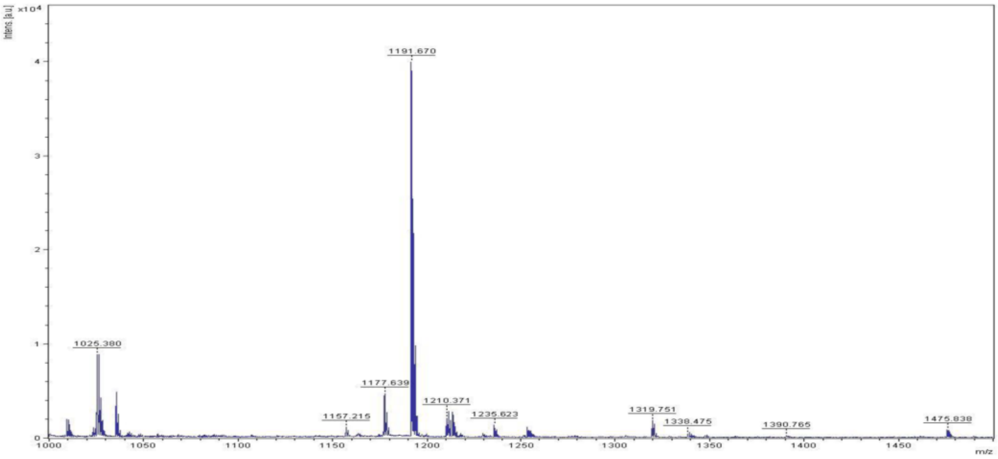

B

Scrambled peptide

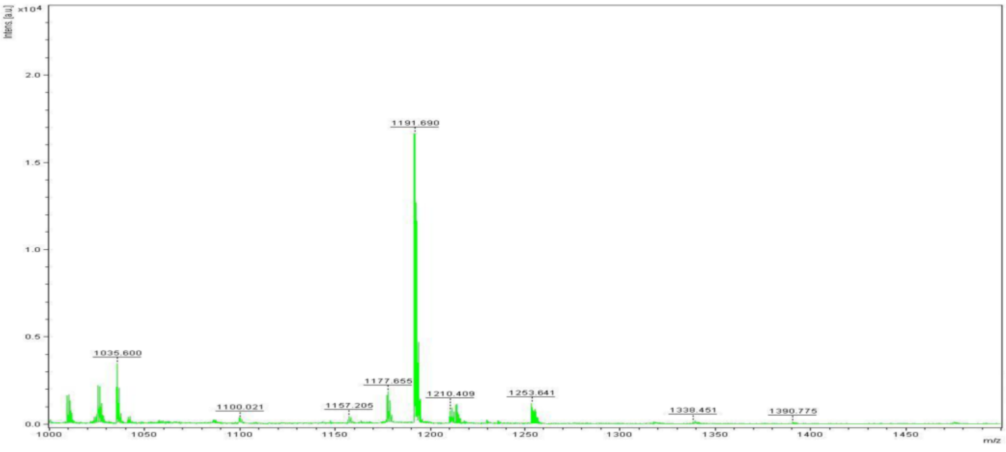

C

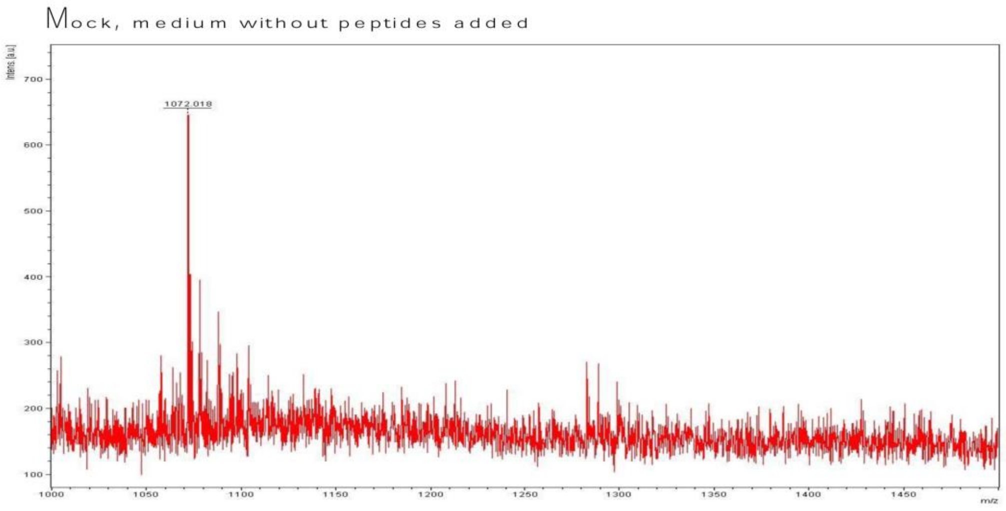

Supplementary Figures S7A-C. Stability of scrambled and 7-13 Fhit peptides evaluated by MALDI-TOF. Twenty-four hours after their administration to the medium, both 7-13 Fhit (A) and scrambled (B) peptides resulted still stable and undegraded. Medium without peptides added (C) was taken as control.

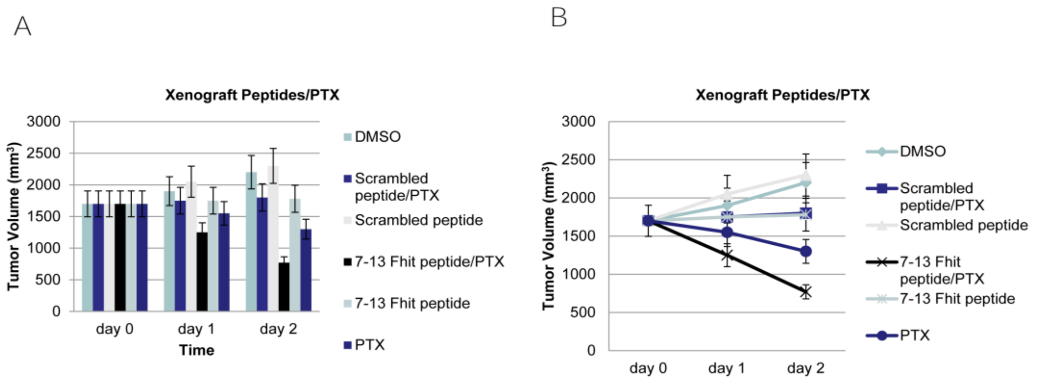

Supplementary Figures S8A-B. Tat-Fhit 7-13 peptide in combination with paclitaxel blocks *in vivo* tumor formation. Nude mice were subcutaneously injected with 1.5 x 10^7 A549 cells. When tumors reached 15 mm diameter, mice were mock-treated, treated paclitaxel (single IV administration of 40 mg/kg), with scrambled Tat peptide (50 mg/kg) alone or in combination with paclitaxel, with Tat-Fhit 7-13 peptide (50 mg/Kg) alone or in combination with paclitaxel; paclitaxel and peptides were injected in a single admnistration. Tumor volume was monitored on daily basis and data is reported as histogram (A) and as lines (B). Bar graphs show mean ± SEM for values from 5 mice (* P < 0,05).
